# Repair of left ventricular driveline tear in a SynCardia-total artificial heart patient

**DOI:** 10.1186/1749-8090-9-7

**Published:** 2014-01-07

**Authors:** Sotirios Spiliopoulos, Magda Tenderich, Dilek Guersoy, Guenes Dogan, Reiner Koerfer, Gero Tenderich

**Affiliations:** 1 Department for the Surgical Therapy of End-stage Heart Failure and Mechanical Circulatory Support, Heart and Vascular Center Duisburg, Fahrner street 135-137, Duisburg 47169, Germany

**Keywords:** Artificial heart, Cardiac failure, Heart assist devices

## Abstract

We report the case of a 64-year old Caucasian male patient with a tear of the left ventricular driveline just above the driveline-air tube junction. We describe the repair technique and the necessary set of tools.

## Background

Tear of a SynCardia-Total Artificial Heart driveline is a rare but potentially life-threatening event. Once it has been identified it has to be sufficiently repaired, otherwise air leak would result into pressure drop, malpositioning of the diaphragm and consequently rapid decrease of artificial ventricle fill volume.

## Case presentation

The SynCardia CardioWest TAH is a pneumatically driven, biventricular system used for the orthotopic replacement of the native heart ventricles in cases of end-stage heart failure and refractory cardiogenic schock [1,2]. Two wire-reinforced, dacron-coated drivelines connect the artificial ventricles to the pneumatic drivers of the external console. Like all extracorporeal components, these drivelines are prone to wear and tear. We describe the repair technique of a left ventricular driveline tear in a SynCardia Total Artificial Heart patient and present the set of tools used.

**Figure 1:**
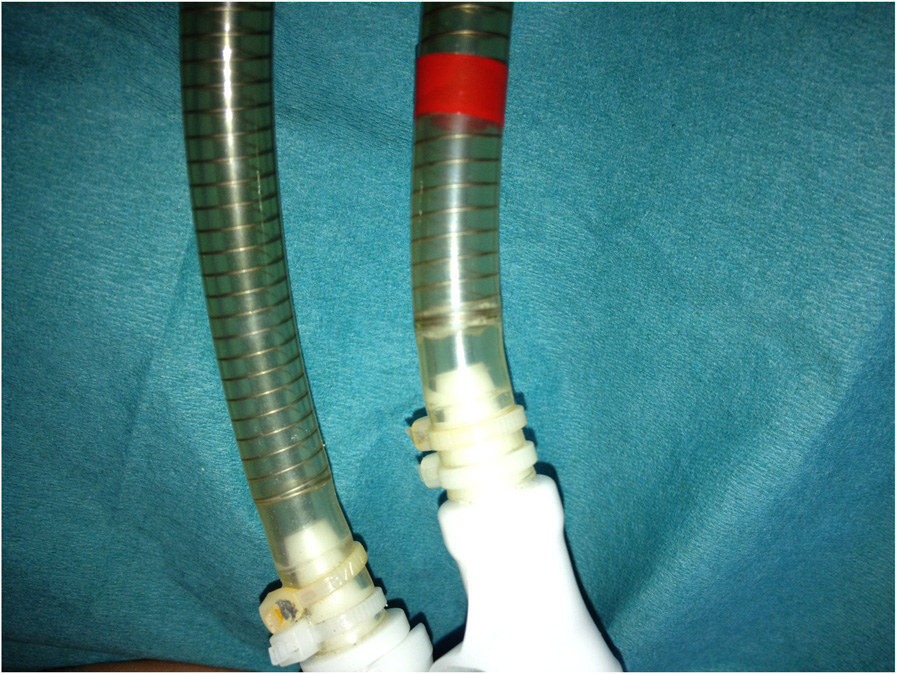
Small horizontal tear at the left ventricular driveline.

**Figure 2:**
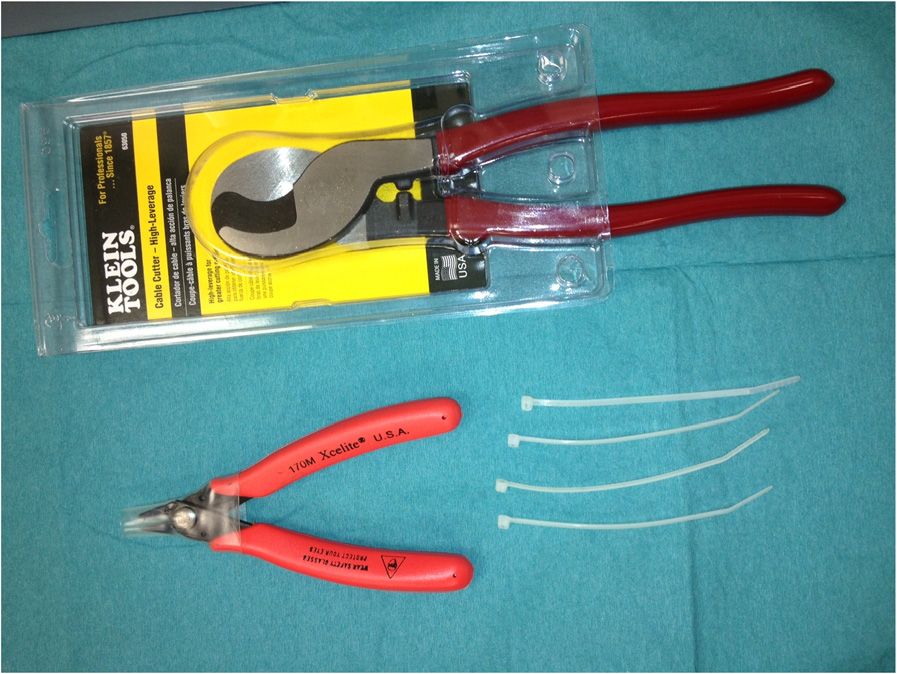
Set of tools: Xcelite 170 M general purpose shear cutter (Apex tool group, Sparks, MD, USA), high leverage cable cutter (Klein Tools, Lincolnshire, IL, USA), cable ties.

**Figure 3:**
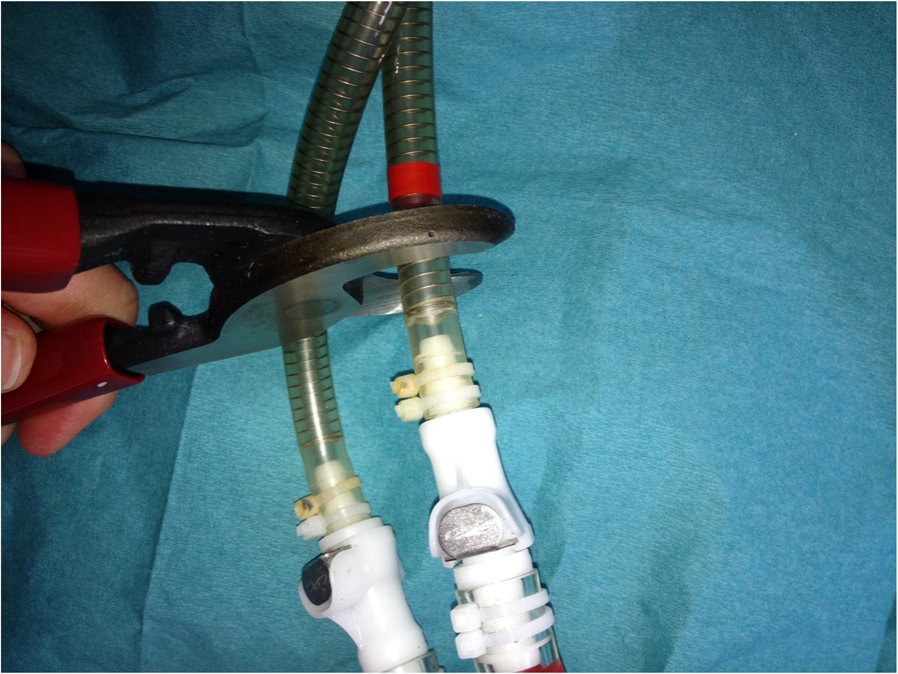
Cutting off the left driveline with the high leverage cable cutter.

A 64-years old male, caucasian patient was admitted for therapy of a refractory cardiogenic schock on the grounds of an ischemic cardiomyopathy. Despite innotropic therapy the patient continued to decline. Initially we implemented veno-arterial extracorporeal membrane oxygenation as a bridge to decision and finally implanted the 70-cc SynCardia-TAH as a bridge to transplantion. The postoperative course was uneventful. The patient was switched to the Freedom driver and was discharged at home. The patient was readmitted at postoperative day 172 due to progressive exertion intolerance caused by an air leak at the left ventricular driveline. Radiology showed mid-severe pulmonary congestion. Upon inspection, we noted a small horizontal tear above the connection of the driveline to the air tubes (Figure 1). After cutting through both cable ties that secure the drivelines with the connectors using a Xcelite 170 M general purpose shear cutter (Apex tool group, Sparks, MD, USA) (Figure 2) we cut off the left driveline slightly above the location of the tear with a high leverage cable cutter (Klein Tools, Lincolnshire, IL, USA) (Figure 3) and quickly switched the patient to a Companion Driver operating in a standard Intensive Care Unit Mode. The whole procedure was completed in approximately 5 minutes. Throughout the procedure the patient showed no clinical signs such as syncopal spell. Two days later the patient recompensated fully. He was switched to a Freedom Driver and discharged at home. Mechanical support of the patient is still ongoing.

## Conclusions

Driveline tear is a rare event that potentially harbours the risk of insufficient filling of the artificial ventricles. In our case patient became symptomatic in terms of pulmonary congestion and progressive exertion intolerance. Therefore it is important to identify and repair quickly the defect. Other currently available repair techniques include banding the driveline with a silicon tube [3] or sealing it with cyanoacrylate ashesive. However this is only a provisory repair. Since the U.S. Food and Drug Administration (FDA) has approved a Humanitarian Use Device (HUD) designation for the SynCardia temporary Total Artificial Heart to be used for destination therapy in patients not being eligible for transplantation [4] and since the durability of the driveline could limit successful long-term support [5], the aim should be to definitively repair any driveline defect As in this case, the repair can be easily facilitated by cutting the driveline at the location of the tear and reconnecting to the air tubes. During repair special care should be taken to avoid any low-output events. For this reason repair should be performed by a team of two physicians who are responsible for cutting the driveline and switching the patient to the new Driver and one technician who supervises the Driver settings. Furthermore we would recommend in a first step to switch the patient to a Companion Driver in order to control fill volumes, pumping rate and pressure and finally switch to a Freedom Driver.

## Consent

Written informed consent was obtained from the patient for publication of this case report and any accompanying images. A copy of the written consent is available for review by the Editor-in-Chief of this journal.

## Abbreviations

TAH: Total artificial heart; HUD: Humanitarian use device; FDA: Food and drug administration.

## Competing interests

The authors declare that they have no competing interests.

## Authors’ contributions

SS drafted the manuscript. MT was involved in the drafting of the manuscript. DG was involved in the drafting of the manuscript. GD revised the manuscript. RK has given final approval of the version to be published. GT has given final approval of the version to be published. All authors read and approved the final manuscript.
